# Supplementary material for: Low-noise GaAs quantum dots for quantum photonics
Source: Nat Commun. 2020 Sep 21;11:4745. doi: 10.1038/s41467-020-18625-z (PMC7506537; doi:10.1038/s41467-020-18625-z)
Supplement: Supplementary file 1 — Supplementary Information [file 41467_2020_18625_MOESM1_ESM.pdf]

Supplementary Information

Low-noise GaAs Quantum Dots for Quantum Photonics

Liang Zhai,[1] Matthias C. Löbl,[1] Giang N. Nguyen,[1,2] Julian Ritzmann,[2] Alisa Javadi,[1] Clemens Spinnler,[1] Andreas D. Wieck,[2] Arne Ludwig,[2] and Richard J. Warburton[1]

[1]*Department of Physics, University of Basel, Klingelbergstrasse 82, CH-4056 Basel, Switzerland*

[2]*Lehrstuhl für Angewandte Festkörperphysik, Ruhr-Universität Bochum, DE-44780 Bochum, Germany*

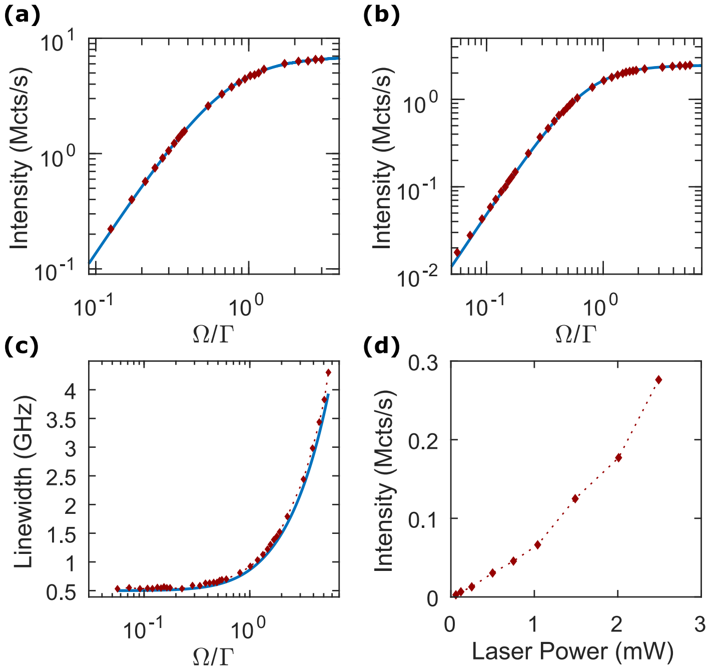

Supplementary Figure 1. **Power dependent measurements on QD1 and QD2.** **(a)** Intensity of the resonance fluorescence (red diamonds) of X^{1-} from QD1 as a function of the normalised Rabi-frequency Ω/Γ. The resonance fluorescence is measured using a dark-field confocal microscope[1]. The intensity saturates at around 6.5 Mcts/s (raw count-rate). The blue curve is a theoretical fit of a two-level model[2]. **(b)** Power dependent resonance fluorescence measurement (red diamonds) of X^{1-} from QD2. The measurement is performed similar to the one shown in (a) and recorded with a superconducting nanowire single-photon detector. The intensity saturates around 2.5 Mcts/s (raw count-rate). The blue curve represents again a theoretical fit of a two-level model. **(c)** Optical linewidth (red) of the resonant fluorescence from QD2 X^{1-} displayed as a function of the normalised Rabi-frequency Ω/Γ. The linewidth measurements are performed by scanning the gate voltage acorss the QD resonance under different excitation laser powers (the laser frequency is fixed). The linewidths are fitted to Lorentzian functions and converted into frequency unit using a Stark shift of 621.679 GHz/V (see Fig. 2(c) of the main text). The linewidths stay very close to the lifetime limit (496 MHz) at low power and become broader due to power broadening at higher power. The blue curve represents the power broadening effect of a two-level system. The plot is not a fit but just the theoretical model using the parameters Γ as well as Ω extracted from (b). The measured optical linewidths stay close (marginally above) to theoretical values for all excitation powers. **(d)** Intensity of photoluminescence of X^{1-} from the same QD, plotted as a function of non-resonant laser power. This non-resonant laser is a CW He-Ne laser emitting at $\lambda = 632.8$ nm. The non-resonant laser power is measured before we send it to the confocal microscope. Photoluminescence is measured at the same gate voltage as in (b). It is collected by the microscope setup, sent through a grating-based filter (50 GHz bandwidth, for filtering out non-resonant laser) and counted by the superconducting nanowire single-photon detector. Under lower-power non-resonant excitation, we determine an upper bound of 7.7 GHz for the photoluminescence linewidth. This boundary is obtained by fitting the photoluminescence spectrum to a Lorentzian function, and is limited by the resolution of the spectrometer. The actual photoluminescence linewidth could be much narrower.

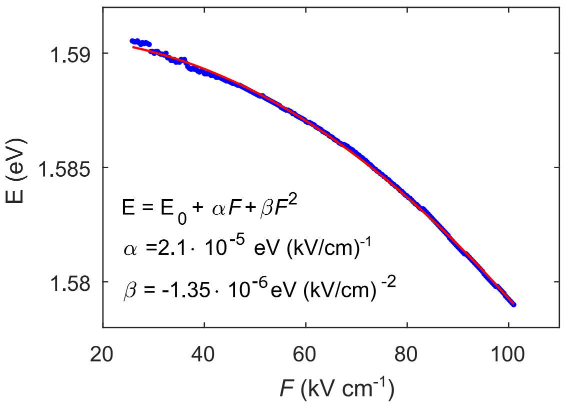

Supplementary Figure 2. **Photoluminescence as a function of electric field.** Photoluminescence energy (E) versus electric field[3] (F) for the quantum dot shown in Fig. 1 of the main text. The electric field is obtained by a bandstructure simulation. The solid red curve is a quadratic fit to the data. We extract the permanent dipole moment to be $\alpha/e = 0.21$ nm, and the polarisability $\beta = -1.35\ \mu$eV (kV/cm)$^{-2}$.

.

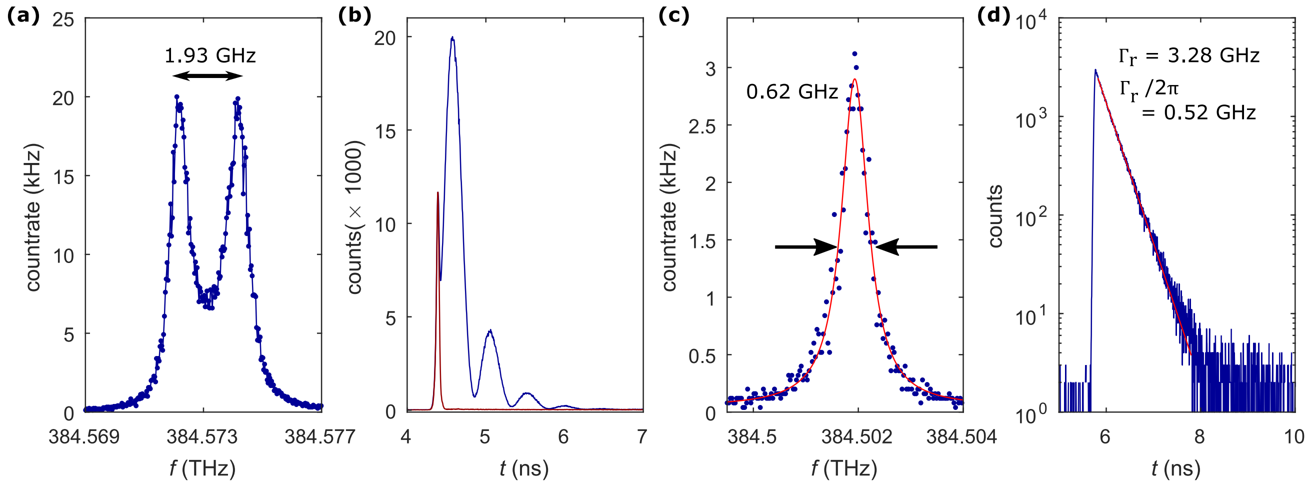

Supplementary Figure 3. **Resonant linewidth and lifetime measurements on X^0 and X^+.(a)** Resonance fluorescence from the neutral exciton, X^0, measured on QD2. The neutral exciton has a small fine structure splitting of FSS = 1.93 GHz (7.98 μeV). **(b)** Lifetime measurement on the X^0 (QD2). Resonance fluorescence (blue) is measured as a function of the time delay t after exciting X^0 with a picosecond laser-pulse. Here, a pronounced quantum beat with a frequency of $\frac{2\pi}{\mathrm{FSS}}$ is observed. The red curve corresponds to the background from the scattered laser light. **(c)** Resonance fluorescence measurement (blue) on the positively charged exciton, X^+, from QD2. The red curve is a Lorentzian fit. The X^+ shows a narrow optical linewidth of 0.62 GHz. **(b)** Lifetime measurement (blue) on the X^+ (QD2). The radiative decay rate, which corresponds to a natural linewidth of $\Gamma_r/2\pi = 0.52$ GHz, is extracted by fitting an exponential curve (red).

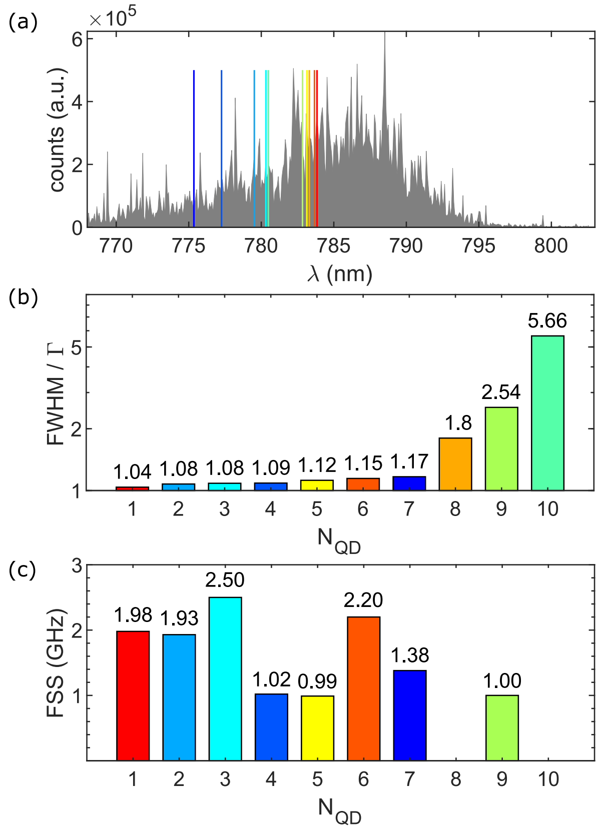

Supplementary Figure 4. **Summary of the optical properties of ten randomly chosen QDs.** **(a)** Emission of the QD-ensemble at a gate voltage of $V_\mathrm{g} = -0.4$ V. The coloured lines indicate the emission wavelengths of the QDs (X^{1-}) which have been measured in detail. **(b)** The ratio between measured optical linewidth (full width at half maximum, FWHM) on the negatively charged exciton X^{1-} and its lifetime limit (Γ) shown for ten randomly chosen QDs. On the x-axis, $\mathrm{N_{QD}}$ indicates the QD number sorted by the ratio FWHM/Γ in the ascending order. The colours of the bars are linked to the colours in (a). In the ideal case, the optical linewidth of QD reaches the lifetime limit: the ratio FWHM/Γ is one. For the majority of QDs, the ratio is close to one – below a level of FWHM/$\Gamma = 1.2$ we find seven QDs out of ten. These QDs suffer from little noise. The QD1 and QD2 investigated in the paper are labelled here as $\mathrm{N_{QD}} = 3$ and $\mathrm{N_{QD}} = 2$, respectively. For few QDs, there is a rather large broadening of the linewidth beyond the lifetime limit. The lifetime limits of QD1, QD4 - QD10 are 510 MHz, 640 MHz, 515 MHz, 520 MHz, 437 MHz, 496 MHz, 250 MHz, 530 MHz, respectively. **(c)** Fine structure splitting (FSS) for the neutral exciton (X^0) measured on the same QDs as in (b). The FSS is determined by scanning the laser frequency across the QD resonance. An example is shown in Supplementary Figure 3. As in (b), the colour of the bars is linked to the QD wavelengths in (a). For most of the QDs, the FSS is below 2 GHz. For $\mathrm{N_{QD}} = 9$, we state here an upper bound of 1 GHz for its FSS. For $\mathrm{N_{QD}} = 8$ & 10, determining the FSS by scanning a laser across X^0 was not successful due to the relatively large linewidth.

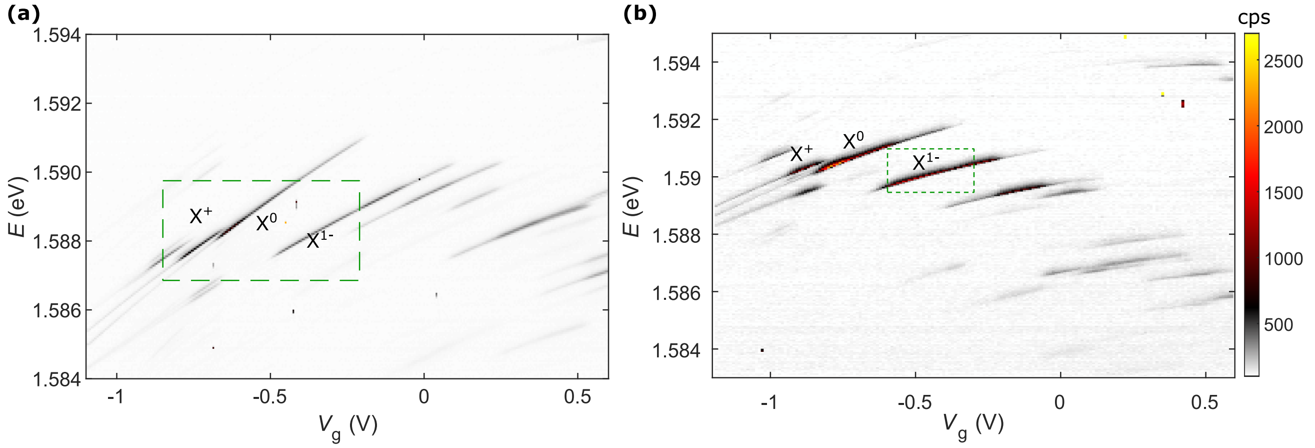

Supplementary Figure 5. **Photoluminescence charge plateaus of QD1 and QD2.** **(a)** Charge plateaus of QD1 measured in photoluminescence. We observe emission over a wide range of excitons with different net-charges. The green dashed frame indicates the scan-range of the measurement in Fig. 1(c) of the main text. **(b)** Similar photoluminescence measurement on QD2. Again, several charge plateaus are observed. The green dashed area indicates the scan-range of the resonance fluorescence measurement in Fig. 2(c) of the main text.

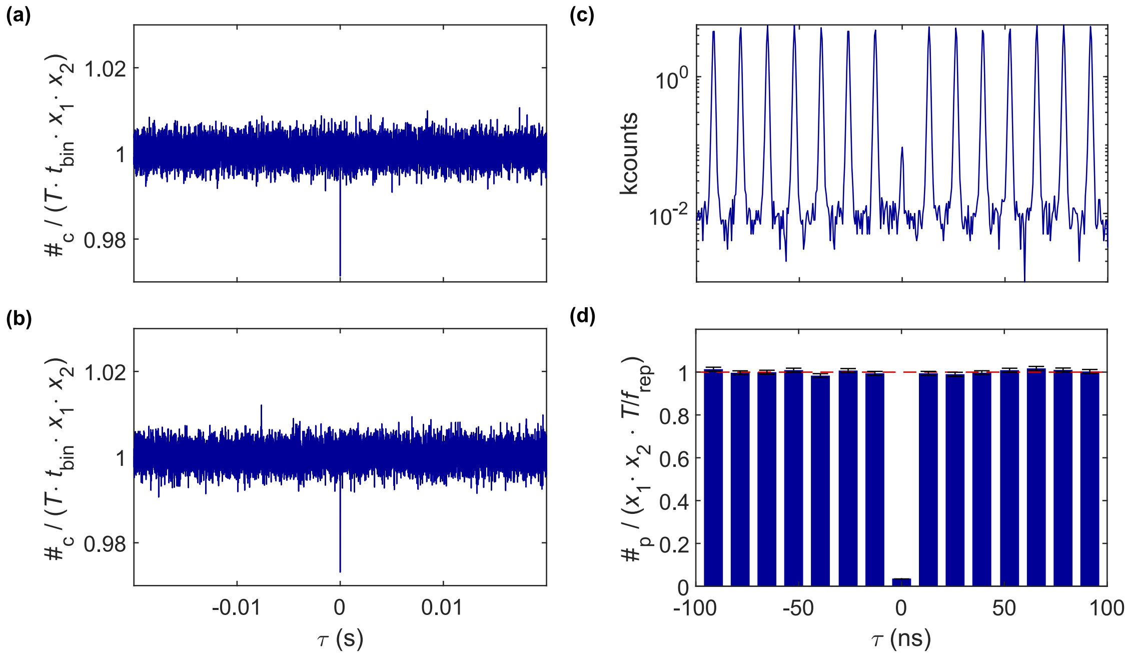

Supplementary Figure 6. **Auto-correlation of the QD1 emission ($\mathbf{X}^{1-}$) from s-to-s recombination for non-resonant and pulsed excitation.** **(a)** The auto-correlation measurement performed under above-band excitation with a laser at $\lambda = 632.8$ nm. The $g^{(2)}(\tau)$ is close to the ideal Poissonian limit of one for all time-scales, demonstrating the long-time stability of the emitter. **(b)** The auto-correlation measurement performed by exciting the quantum dot via p-shell excitation. Similarly here, the $g^{(2)}$ is flat and close to the ideal Poissonian limit. **(c)** The pulsed $g^{(2)}$-measurement (resonant π-pulse excitation) from Fig. 2(e) plotted on a logarithmic scale and evaluated on a longer time-scale. The offset (about ten coincidence events) arises from detector dark counts. For the calculation of the $g^{(2)}(0)$ value in the main text ($g^{(2)}(0) = 0.019$), we have subtracted this dark-counts induced background. By integrating all coincidence events over one full pulse period without substrating the background, we estimate a "worst-case" upper limit: $g^{(2)}(0) = 0.036$. **(d)** The same $g^{(2)}$-measurement as in (c) but plotted as a histogram. To obtain the histogram, we sum up all coincidence events within every single pulse and normalise it by the expectation value for the coincidence events in the case of an ideal Poissonian source: $\langle \#_{\rm p} \rangle = x_1 x_2 T_{\rm int}/f_{\rm rep}$, where x_1 and x_2 are the count rates of each detector channel, $T_{\rm int} = 2500$ s is the overall integration time for the measurement, and $f_{\rm rep} = 76.36$ MHz is the repetition rate of the pulsed laser. Using this normalisation factor, the $g^{(2)}$-measurement normalises to a value very close to the ideal limit of one. This evaluation shows that the quantum dot is a very stable emitter under resonant π-pulse excitation. The normalisation factor $\langle \#_{\rm p} \rangle$ is obtained by a similar consideration compared to the case of continuous-wave excitation (see Ref. 4): let p_1 and p_2 be the probabilities that a photon is detected on channel 1 or 2 after the π-pulse excitation. The count-rates x_1, x_2 are then connected to these probabilities by $x_1 = f_{\rm rep} p_1$, $x_2 = f_{\rm rep} p_2$. In case of two uncorrelated channels, the joint probability for one detection event on channel 1 together with another detection event for a later or earlier excitation pulse on channel 2 is $p_1 p_2 = x_1 x_2 / f_{rep}^2$. The expectation value for the overall number of joint (coincidence) events is then $p_1 p_2$ times the overall number of π-pulses, $T_{\rm int} \cdot f_{\rm rep}$. This consideration leads to the aforementioned expression: $\langle \#_{\rm p} \rangle = x_1 x_2 / f_{\rm rep}^2 \times T_{\rm int} \cdot f_{\rm rep} = x_1 x_2 T_{\rm int}/f_{\rm rep}$.

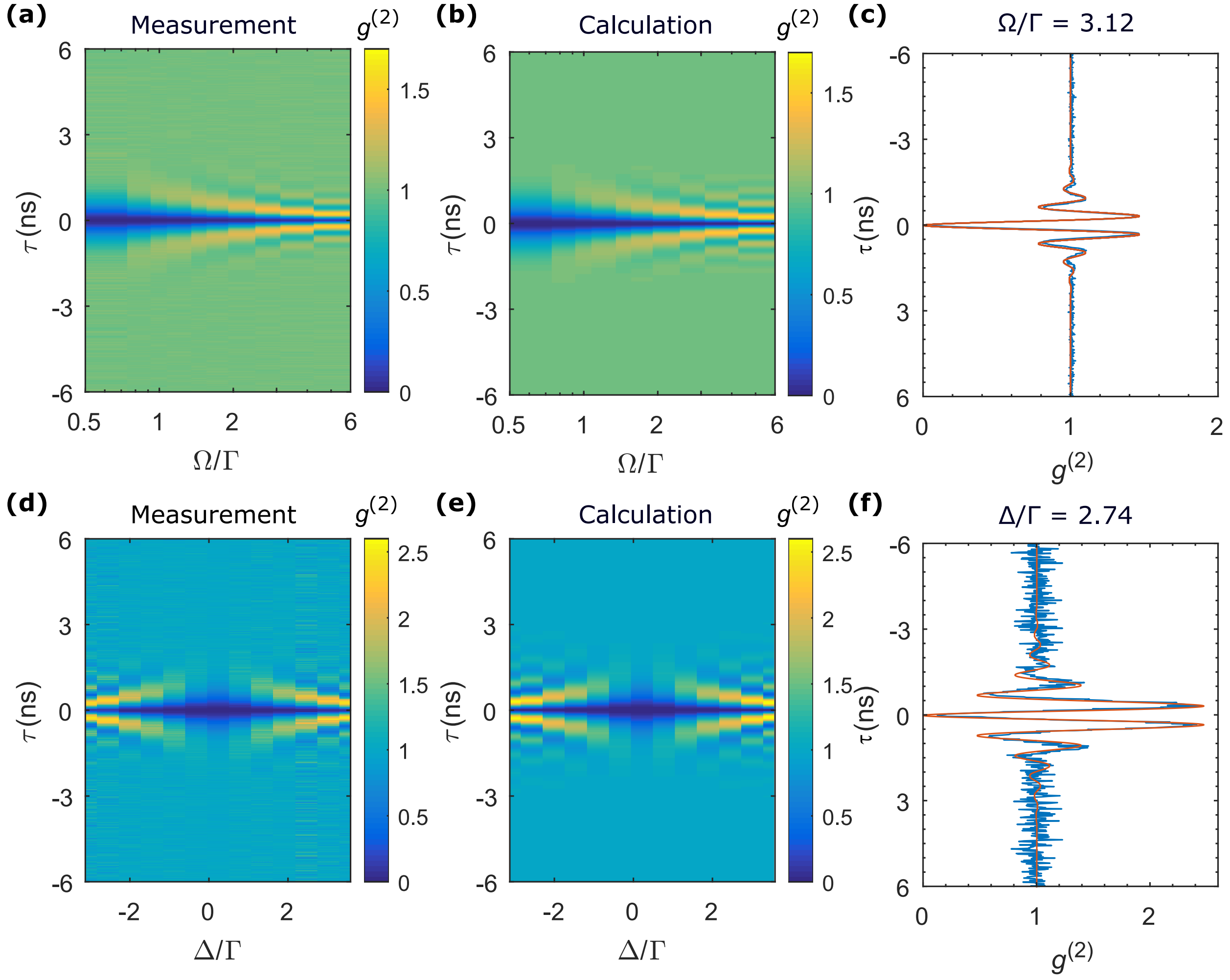

Supplementary Figure 7. **Power-dependent and detuning-dependent autocorrelation measurements.** **(a)** Time-resolved intensity autocorrelation measurement, $g^{(2)}(\tau)$, as a function of the normalised Rabi-frequency Ω/Γ. The measurement is performed under continuous wave (CW) resonant excitation on the negatively charged exciton, X^{1-}, from QD2. The $g^{(2)}(\tau)$ is normalised to one by the number of coincidence events at 300 ns delays. The Rabi frequency Ω is extracted independently from a power saturation curve under CW resonant excitation, while the radiative decay rate Γ is obtained by fitting an exponential function to the lifetime measurement. **(b)** Calculation of the power-dependent autocorrelation function. The $g^{(2)}(\tau)$ function is calculated by solving the optical Bloch equations of a two-level system and then applying the quantum regression theorem. The calculation is carried out with QuTip[5]. We chose to ignore upper-level dephasing in this calculation[6]. The normalised Rabi-frequency is taken from the measurement in **(a)**. Under a strong driving field ($\Omega \gg \Gamma$), the $g^{(2)}(\tau)$ value approaches its upper bound[7] $g^{(2)}(\tau = \pm\pi/\Omega) = 2$. **(c)** Comparison between the measured $g^{(2)}(\tau)$ (blue) and the calculation (red) for $\Omega/\Gamma = 3.12$. **(d)** Time-resolved intensity autocorrelation measured as a function of normalised laser-detuning, Δ/Γ. The excitation laser power is locked to $\Omega = 0.49\ \Gamma$. **(e)** Calculation of the detuning-dependent autocorrelation function in a two-level system. Under a detuned driving, the effective Rabi-frequency[2,8] is represented as $\sqrt{\Omega^2 + \Delta^2}$. In the calculation, the dephasing is again set to zero, and the values of Δ/Γ are taken from (d). In both the measurement and the calculation, the maximum value of $g^{(2)}(\tau)$ exceeds 2, the upper bound in the resonant case, when Δ is relatively large compared to Γ. **(f)** Comparison between the experiment (blue) and the calculation (red) under the condition $\Delta/\Gamma = 2.74$, $\Omega/\Gamma = 0.49$. We find a very good overlap between the data and the calculation curve, indicating the X^{1-} behaves here as an ideal two-level system.

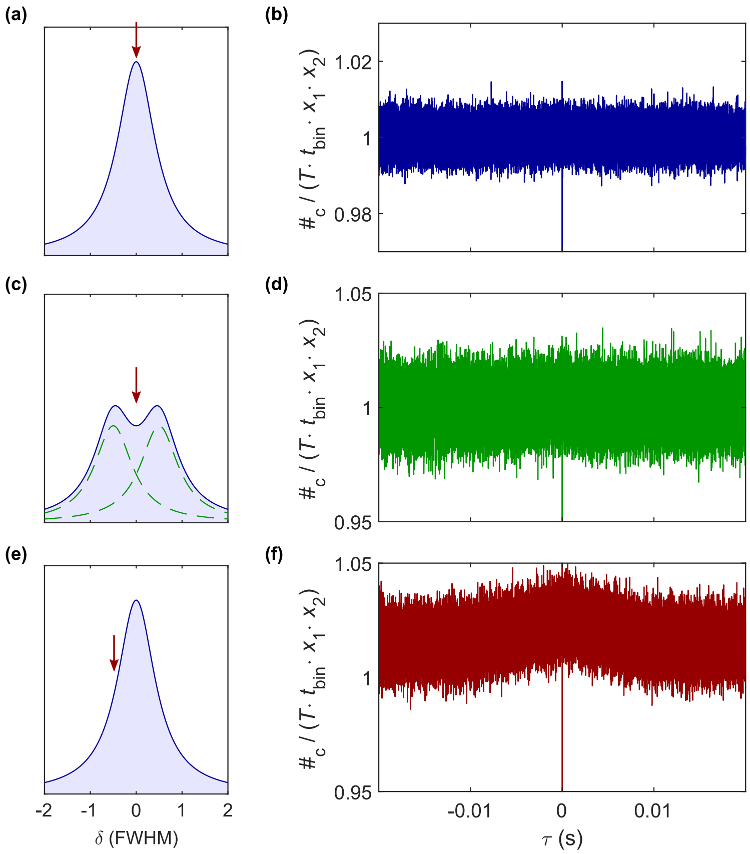

Supplementary Figure 8. **Auto-correlation of the resonance fluorescence on QD1 X^{1-}.** **(a)** Configuration for resonant auto-correlation measurement. The measurement is performed at zero magnetic field ($B = 0$ T) with the laser on resonance with the quantum dot ($\delta = 0$). **(b)** Result of the auto-correlation measurement, $g^{(2)}(\tau)$, as described in (a) evaluated for long time-scales. The $g^{(2)}(\tau)$ is perfectly flat and stays close to one. **(c)** Configuration for an auto-correlation measurement with enhanced sensitivity to spin noise. This measurement is performed at a finite magnetic field ($B = 20$ mT) along the growth direction with the laser frequency centred between the two Zeeman peaks. **(d)** Result of the $g^{(2)}$-measurement as described in (c) for long time-scales. The $g^{(2)}(\tau)$ remains flat and close to one. **(e)** Configuration for an auto-correlation measurement with enhanced sensitivity to charge noise. This measurement is performed at zero magnetic field with the laser slightly detuned (by about half of the linewidth) with respect to the quantum dot resonance. **(f)** Result of the $g^{(2)}$-measurement as described in (e). Here, a bunching on a millisecond time-scale can be seen, showing that some charge noise is present. The charge noise is likely to account for the residual linewidth broadening of X^{1-}.

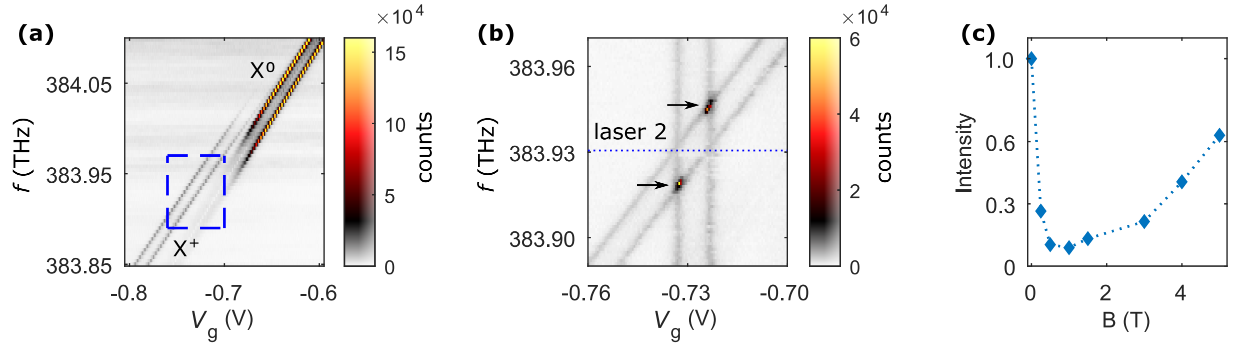

Supplementary Figure 9. **Spin pumping on the positively charged trion.(a)** Resonance fluorescence charge-plateau of the positively charged trion, X^{1+}, and the edge of the X^0 charge-plateau (QD1). The X^{1+} lines are split in a magnetic field ($B = 1.5$ T). The resonance fluorescence is weak due to optical spin-initialisation of the hole spin. **(b)** The signal recovers (marked with arrows) on addressing a second spin ground state with a second laser (dashed line). This second laser is kept at a fixed frequency and a fixed power (same power as in (a)). The blue frame in (a) indicates the range over which the gate voltage and the first laser is tuned in this measurement. Two additional vertical lines are observed when the fixed laser is on resonance with the two vertical transitions. **(c)** The brightness of the X^{1+} resonance fluorescence as a function of the magnetic field. The brightness is normalised to the resonance fluorescence intensity at $B = 0$ T. At about 1 T, the signal has a minimum, suggesting that the lifetime of the hole-spin is the longest at this magnetic field[9].

[1] Kuhlmann, A. V. *et al.* A dark-field microscope for background-free detection of resonance fluorescence from single semiconductor quantum dots operating in a set-and-forget mode. *Rev. Sci. Instrum.* **84**, 073905 (2013).

[2] Loudon, R. *The quantum theory of light* (OUP Oxford, 2000).

[3] Warburton, R. J. *et al.* Giant permanent dipole moments of excitons in semiconductor nanostructures. *Phys. Rev. B* **65**, 113303 (2002).

[4] Löbl, M. C. *et al.* Radiative Auger process in the single-photon limit. *Nat. Nanotechnol.* **15**, 558–562 (2020).

[5] Johansson, J. R., Nation, P. D. & Nori, F. Qutip 2: A python framework for the dynamics of open quantum systems. *Computer Physics Communications* **184**, 1234–1240 (2013).

[6] Jahn, J.-P. *et al.* An artificial Rb atom in a semiconductor with lifetime-limited linewidth. *Phys. Rev. B* **92**, 245439 (2015).

[7] Flagg, E. B. *et al.* Resonantly driven coherent oscillations in a solid-state quantum emitter. *Nat. Phys.* **5**, 203 (2009).

[8] Rezai, M., Wrachtrup, J. & Gerhardt, I. Detuning dependent Rabi oscillations of a single molecule. *New J. Phys.* **21**, 045005 (2019).

[9] Dreiser, J. *et al.* Optical investigations of quantum dot spin dynamics as a function of external electric and magnetic fields. *Phys. Rev. B* **77**, 075317 (2008).
